# Assessing water status in rice plants in water-deficient environments using thermal imaging

**DOI:** 10.1186/s40529-025-00452-4

**Published:** 2025-01-26

**Authors:** Chin-Ying Yang, Yan-Ci Zhang, Ya-Ling Hou

**Affiliations:** 1 Department of Agronomy, National Chung Hsing University, Taichung, 402 Taiwan; 2 Smart Sustainable New Agriculture Research Center (SMARTer), National Chung Hsing University, Taichung, 402 Taiwan; 3 Innovation and Development Center of Sustainable Agriculture (IDCSA), National Chung Hsing University, Taichung, 402 Taiwan; 4 Crop Science Division, Taiwan Agricultural Research Institute, Ministry of Agriculture, Taichung, 413 Taiwan

**Keywords:** Rice, Drought, Thermal imaging, Crop water stress index, Smart agricultural

## Abstract

**Background:**

Rice is a staple food for the global population. However, extreme weather events threaten the stability of the water supply for agriculture, posing a critical challenge to the stability of the food supply. The use of technology to assess the water status of rice plants enables the precise management of agricultural water resources.

**Results:**

The results of this study reveal that rice-producing regions with more severe drought have higher ion leakage rates, lower Soil Plant Analysis Development (SPAD) meter values, and reduced total chlorophyll content in plants. Although no significant differences were observed in red-green-blue (RGB) images, physiological parameters and canopy temperature differed significantly from conventional farming when infrared thermography was used to capture rice plants in the early stages of drought. The Crop Water Stress Index (CWSI), calculated from canopy temperature and atmospheric temperature, indicated a high correlation between access to water for rice plants and their physiological parameters. Regression analysis using CWSI and plant water status yielded a corrected coefficient of determination of 0.86.

**Conclusion:**

Our study demonstrate that infrared thermography can effectively detect early signs of water stress in rice, aiding farmers in irrigation planning and enabling precise management and optimal utilization of water resources.

**Supplementary Information:**

The online version contains supplementary material available at 10.1186/s40529-025-00452-4.

## Introduction

Substantially more food must be produced to feed our rapidly growing global population. In particular, by 2050, food production must increase by 100–110% from current levels to meet the demand (Hussain et al. 2020). Rice (*Oryza sativa* L.) is a crucial food crop. As much as 90% of rice cultivation occurs in Asia, where it serves as a primary food source for the population, providing approximately 30% of the daily caloric intake on average (Ullah et al. 2018; Schneider and Asch 2020; Sultana et al. 2022). In recent years, climate change has increased global average temperatures, resulting in frequent occurrences of extreme weather events such as droughts and floods (Lesk et al. 2016). Rice production also faces challenges due to the depletion of suitable soils for cultivation: Rice yields in tropical regions could decrease by 18–51% by 2100 (Asibi et al. 2019).

Rice crops are subjected to various abiotic stresses throughout their growth and development that may reduce yields, such as high temperatures, floods, and droughts (Jung et al. 2010; Lamaoui et al. 2018). Even in regions with advanced agricultural development and high yields, brief periods of drought or prolonged insufficient rainfall can cause severe drought conditions (Barnabás et al. 2008; Kim et al. 2020b). Studies have demonstrated that rice plants respond to drought stress by curling their leaves to reduce water loss. Curled rice leaves can induce developmental abnormalities, decreasing photosynthetic efficiency, reducing stomatal conductance, and inhibiting transpiration, leading to an increase in leaf temperature (Melandri et al. 2020; Yang et al. 2022).

In response to changing environmental conditions, farmers can incorporate advanced technology and implement precision farming tailored to specific regions with limited resources and land to produce food using minimal resources (Khan et al. 2021). Several studies have utilized infrared thermal imaging technology or digital image analysis to examine plant phenotypic traits and physiological responses (Khanal et al. 2017a; Kim et al. 2020a). In terms of cultivation management, thermal imaging can be employed to evaluate preharvest and postharvest conditions, microbial infection distribution, and produce freshness (Linke et al. 2000). Additionally, by measuring changes in canopy temperature, leaf nitrogen content can be assessed, helping farmers adjust nitrogen application in their fields (Guo et al. 2016). Studies have demonstrated that thermal imaging can detect early differences in leaf temperature caused by pathogen infection and lesion distribution. Such early detection is vital because increases in canopy temperature are associated with water loss (Mahlein 2016; Pipatsitee et al. 2018). Therefore, infrared thermal imaging is a crucial tool for studying the relationship between plant and canopy temperatures and exploring plant physiology and ecology (Jones 2004).

In outdoor field settings, assessments of the current water stressed levels of plants in real-time depend on environmental factors and can be time-consuming. Therefore, scholars employed remote measurement of canopy temperatures using thermal imaging to rapidly provide information and assist in field moisture management (Elsayed et al. 2017). Moreover, Jackson et al. (1981) proposed the concept of the crop water stress index (CWSI) using canopy temperature as an indicator of drought stress. This index has since been widely adopted in assessments of various annual or perennial crops due to its low equipment requirements and suitability for daily field management (Bellvert et al. 2016; Godson-Amamoo et al. 2022).

Large-scale field imaging using drones can assess crop water stress levels under drought conditions; however, drone-based thermal imaging technology has several practical limitations, including high costs and technical requirements. Additionally, drone imaging results are susceptible to environmental conditions such as wind speed, light changes, and temperature fluctuations; strong winds, for instance, can cause instability, reducing image clarity. Drones are also restricted by environmental factors and flight altitude limitations, which can affect imaging quality and applicability. Therefore, the handheld thermal imaging sensor examined in this study provides a cost-effective and easy-to-operate alternative for small-scale farms. It is less affected by factors such as wind speed and flight distance, making it a more flexible and economical choice in certain situations. This study utilized thermal imaging technology to examine rice field moisture management, conducting outdoor drought experiments using the rice variety ‘Tainan 11’(‘TN11’) and assessing physiological changes in the plants and environmental meteorological information to establish correlations with canopy temperature. Rice farmers can utilize the insights from this study to practice advanced water management.

## Materials and methods

### Experimental site and materials

The experimental field was located at the Agricultural Experiment Station of National Chung Hsing University in Taichung, Taiwan (latitude 24.08° N, longitude 120.72° E), an independent agricultural research facility. In this study, the medium-late maturing *japonica* rice variety ‘TN11’was utilized as the experimental material. In Taiwan, rice cultivation typically involves two cropping seasons per year, with one season having a growth period of approximately 121 days and the other having a growth period of approximately 110 days. ’TN11’ is a prevalent variety in Taiwan for its high yield, adaptability, and flavor.

### Experimental field layout and management

Our experiment was conducted using a randomized design, incorporating two water management regimes, conventional plant (CP) and drought treatment. Each treatment plot covered an area of approximately 110 m², with four replicates per water management regime. Rice was planted with a row spacing of 30 cm × 24 cm. Organic compound fertilizer N-P₂O₅-K₂O (16-8-12) was applied at a nitrogen (N) rate of 180 kg ha⁻¹, distributed evenly during three growth stages: early-tillering, mid-tillering, and panicle initiation. Water management treatments were initiated when the rice reached the mid-tillering stage.

The CP plot served as the control group, maintained in a water depth of 1–5 cm until harvest except during the dry field preparation period. By contrast, plants in the drought treatment group were not watered to reduce their water status to a target value, after which conventional irrigation was resumed until harvest.

The drought status of the plots was determined by calculating the water status in the soil. Soil samples (W1) weighing approximately 200 g were randomly collected from a depth of 0 to 10 cm in each treatment plot. These samples were then dried at 80 °C to obtain a dry weight (W2), and the soil water content was calculated. The soil water status was calculated using the following formula:

$${\rm{Relative}}\,{\rm{water}}\,{\rm{status}}\,{\rm{of}}\,{\rm{soil }}\left({\rm{\% }} \right){\rm{ = Soi}}{{\rm{l}}_{\rm{D}}}{\rm{/Soi}}{{\rm{l}}_{{\rm{CP}}}}{\rm{ \times 100\% }}$$

where Soil_D_ represents the soil water content under drought conditions, and Soil_CP_ represents the soil water content under CP conditions.

### Measure of rice plant water status

In this study, the water status in the rice plants were measured to assess the degree to which the plants were affected by drought, with lower values indicating more severe effects of drought. To assess water status, leaf samples from the above-ground portion were cut into 5 cm segments, weighed to determine their fresh weight (FW), soaked in deionized water for 4 h, and then weighed again to determine their turgid weight (TW). Subsequently, the samples were dried at 80 °C until a constant dry weight (DW) was achieved. The relative water content (RWC) of rice plants under both CP and drought conditions was calculated using the following formula:

$${\rm{RWC }}\left({\rm{\% }} \right){\rm{ = }}\left({{\rm{FW - DW}}} \right){\rm{/}}\left({{\rm{TW - DW}}} \right){\rm{ \times 100\% }}$$

and the relative water status of the plant was calculated using the following formula:

$${\rm{Relative}}\,{\rm{water}}\,{\rm{status}}\,{\rm{of}}\,{\rm{plant }}\left({\rm{\% }} \right){\rm{ = RW}}{{\rm{C}}_{\rm{D}}}{\rm{/RW}}{{\rm{C}}_{{\rm{CP}}}}{\rm{ \times 100\% }}$$

where RWC_D_ represents the relative water status of rice plants under drought treatment and RWC_CP_ represents the relative water status of rice plants under CP.

### Determination of chlorophyll content and ion leakage rate

The total chlorophyll content of the rice leaves was measured using both nondestructive and destructive methods. The nondestructive method involved using a chlorophyll meter (Soil Plant Analysis Development [SPAD]-502, Spectrum Technologies, UK) to measure chlorophyll content. In each treatment plot, a random rice plant was selected, and a measuring probe was employed to randomly clamp 5–8 leaves to obtain the SPAD value. Four rice plants were randomly selected for measurement in both the CP and drought conditions plots.

In the destructive method, a random rice plant was selected from each treatment plot for sampling. The above-ground leaves were cut and mixed uniformly, and 1 g of fresh leaf samples was weighed. The samples were then placed in 15 mL centrifuge tubes and mixed with 15 mL of 95% ethanol for extraction for 3 days. After diluting the extract tenfold, absorbance values at wavelengths of 665 nm (A665) and 649 nm (A649) were measured using a spectrophotometer (SpectraMax ABS plus, Molecular Devices, USA) (Wintermans and De Mots 1965).

The method for measuring electrolyte leakage (EL) was adapted from Sedaghat et al. (2017). One rice plant was collected from each treatment plot, and its above-ground leaf blades were cut into 5 cm segments, with a FW of 1 g. These segments were then placed into 50 mL centrifuge tubes, to which 50 mL of deionized water was added. Additionally, a blank group containing only deionized water without leaf segments was prepared. All centrifuge tubes were then placed on a rotary shaker (YIHDER, Taiwan) and shaken at 100 rpm for 3 h. After shaking, the electrical conductivity (EC) of the samples was measured using an EC-400 L conductivity meter (ISTEK, Korea). The samples were then subjected to autoclaving at 121 °C for 20 min and allowed to cool to room temperature before measuring EC again. The EL rate was calculated based on the formula recommended by Jambunathan (2010) and modified as follows:

$${\rm{EL }}\left({\rm{\% }} \right){\rm{ = }}\left({{\rm{E}}{{\rm{C}}_{\rm{1}}}{\rm{ - E}}{{\rm{C}}_{{\rm{Blank1}}}}} \right){\rm{/}}\left({{\rm{E}}{{\rm{C}}_{\rm{2}}}{\rm{ - E}}{{\rm{C}}_{{\rm{Blank2}}}}} \right){\rm{ \times 100\% }}$$

where EC_1_ and EC _Blank1_ represent the EC of the sample and the blank group, respectively, after the first measurement following shaking. EC_2_ and EC _Blank2_ represent the EC of the sample and the blank group, respectively, after the second measurement following high-temperature treatment.

### Canopy temperature analysis and calculation of crop water stress index

To obtain the canopy temperature of the rice plants, thermal imaging was conducted using a handheld thermal camera, Thermo GEAR G100EX (NEC Avio, Japan). At least three images were captured for each treatment. The thermal images and visible light images were imported into TAS infrared thermal analysis software, and 30 temperature points were manually selected on the examined rice plants. The average of these points provided the canopy temperature of the rice plants in each image.

The CWSI was calculated using the formula suggested by Luan et al. (2021):

$${\rm{CWSI = }}\left({{{\rm{T}}_{\rm{c}}}{\rm{ - }}{{\rm{T}}_{{\rm{wet}}}}} \right){\rm{/}}\left({{{\rm{T}}_{{\rm{dry}}}}{\rm{ - }}{{\rm{T}}_{{\rm{wet}}}}} \right)$$

where T_c_ represents the canopy temperature of rice plants under drought conditions, T_wet_ represents the lower limit of the canopy temperature of rice plants under CP and well-watered conditions, and T_dry_ represents the upper limit of the canopy temperature when the stomata are closed and no transpiration occurs.

### Statistical analysis

Statistical analysis was conducted using SAS v. 9.4. First, analysis of variance was employed to assess the relative significance of parameters. Subsequently, the least significant difference (LSD) method was utilized to examine the differences in mean values of parameters, with various letters indicating significant differences (*p* < 0.05). The differences in canopy temperatures between treatments were analyzed using Student’s t-test in Microsoft Excel; an asterisk (*) indicates significant differences between treatments (*p* < 0.05). Additionally, regression analysis was performed to evaluate the relationship between CWSI and plant water status. Pearson correlation analysis of rice plant physiological parameters, canopy temperature, and environmental factors was conducted using R v. 4.2.1.

## Results

### Experimental field setup and rainfall overview

**Fig. 1:**
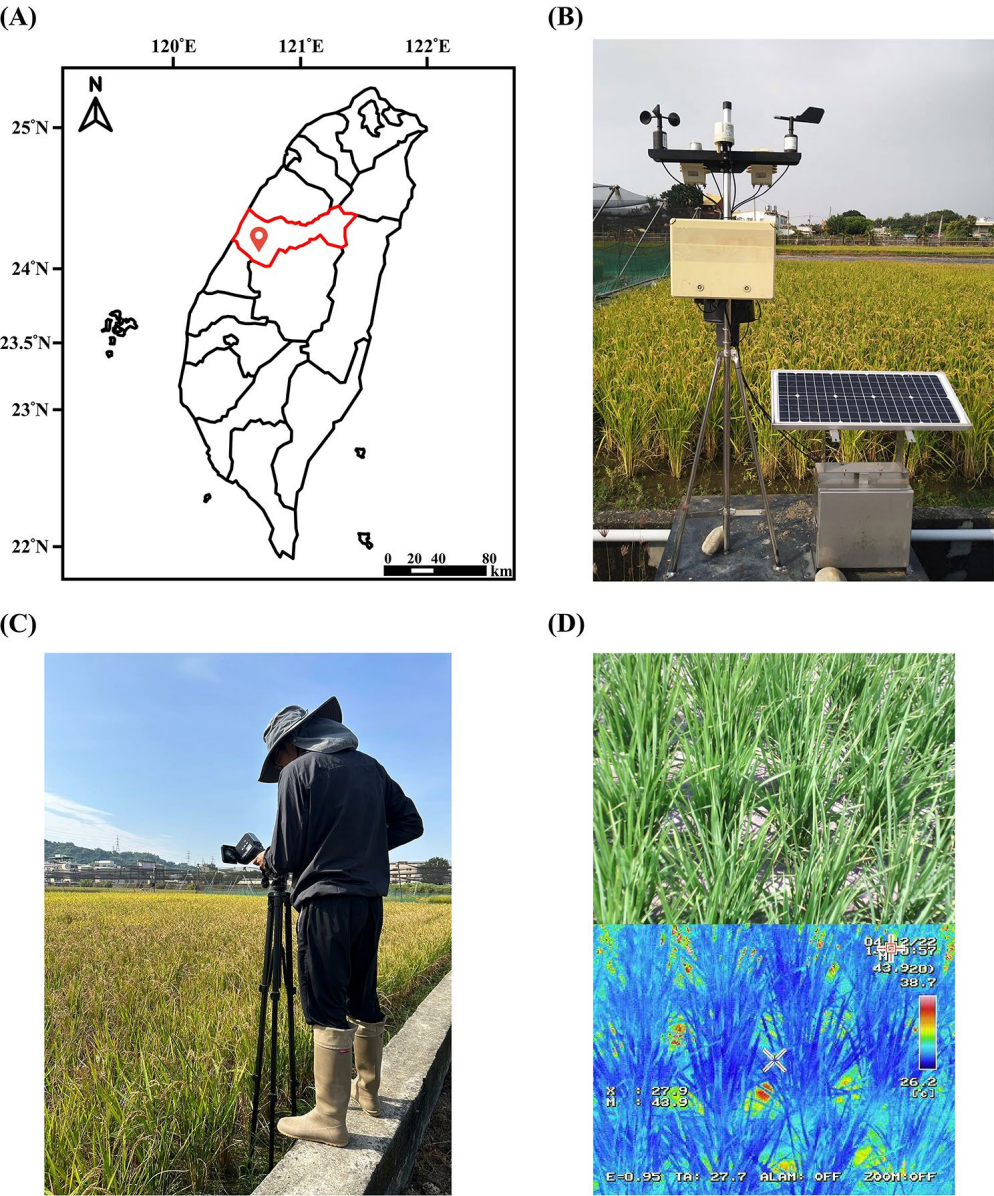
Experimental field location, facilities, and thermal imaging capture. (**a**) The experimental field is located in Wufeng District, Taichung City, Taiwan. (**b**) Micro weather observation station at the edge of the experimental field. (**c**) Image illustrating the process of capturing thermal images using a handheld infrared thermal camera. (**d**) Actual capture of thermal images, with a red-green-blue (RGB) image above and a thermal image below

This study conducted a 1-year rice field experiment in Wufeng District, Taichung City, Taiwan. A micro weather observation station was established at the edge of the experimental site to collect temperature and rainfall data (Fig. 1a, b). During the drought treatment period, thermal images of the rice were captured approximately weekly under favorable weather conditions. The imaging process involved maintaining fixed distance, angle, and parameters, followed by calibration of a handheld thermal camera based on temperature and relative humidity, enabling image capture (Fig. 1c, d).

**Fig. 2:**
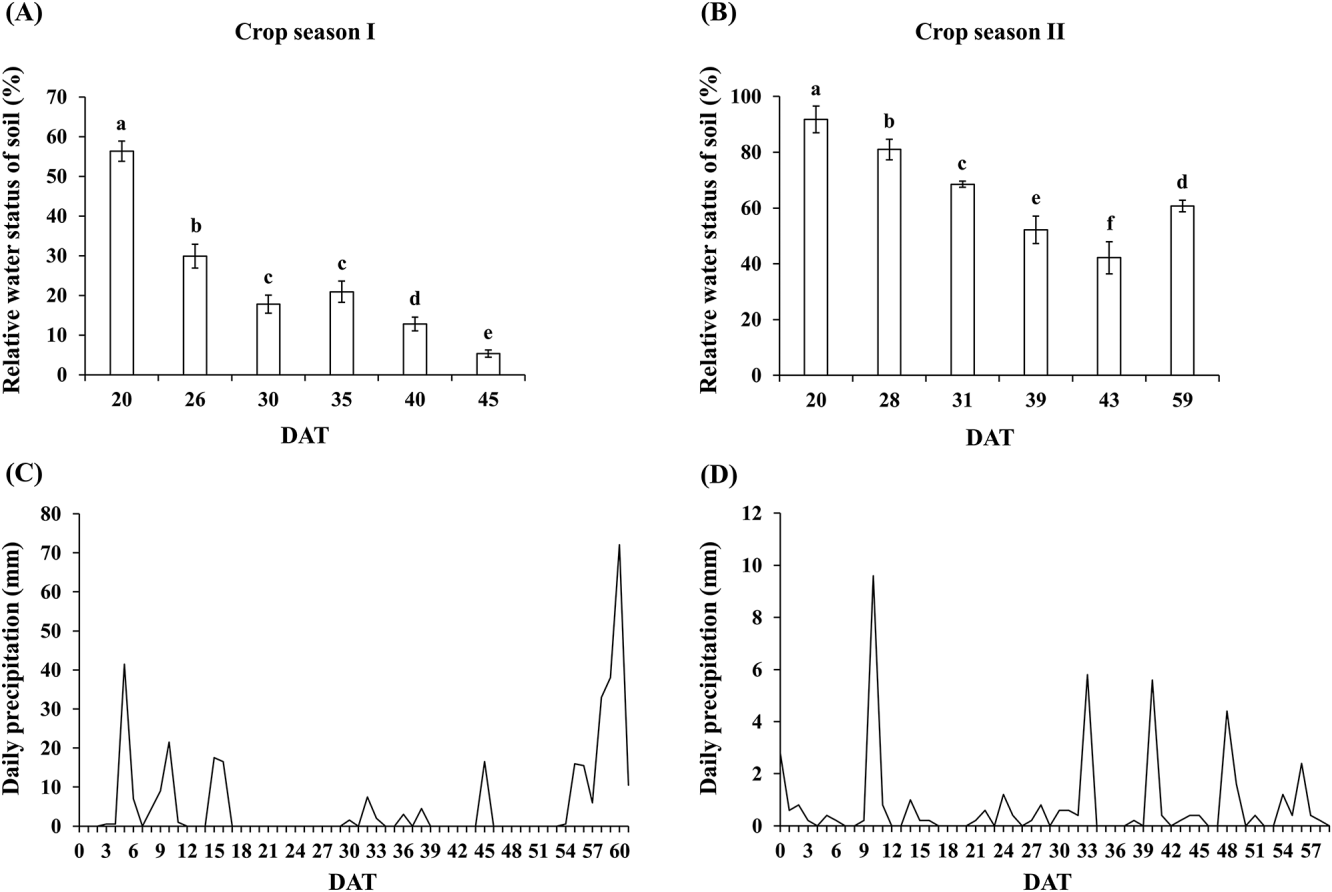
Soil water status and rainfall during drought treatment in the first and second cropping seasons. (**a**) Soil water status during the drought treatment period for the first cropping season of 2022. (**b**) Soil water status during the drought treatment period for the second cropping season of 2022. (**c**) Daily cumulative rainfall during the drought treatment period for the first cropping season of 2022. (**d**) Daily cumulative rainfall during the drought treatment period for the second cropping season of 2022. The experiment was conducted independently for at least three times. Error bars represent standard deviations (SDs). The letters indicate significant differences between samples based on LSD post hoc tests (*p* < 0.05). DAT: day after treatment

The effects of drought treatment in the experimental site were indicated in terms of relative soil moisture content. The results revealed that due to the decreasing rainfall frequency during the mid-stage of the drought treatment in the first cropping season of 2022, the relative soil moisture content in the experimental site fell to as low as 5.3% (Fig. 2a, c). By contrast, during the drought treatment period of the second cropping season, frequent rainfall occurred, resulting in a relative soil moisture content in the experimental site of only 42.2% at its lowest (Fig. 2b, d). Thus, the effectiveness of drought treatment in the field was compromised.

### Physiological changes of ’TN11’ under varying moisture management conditions

**Fig. 3:**
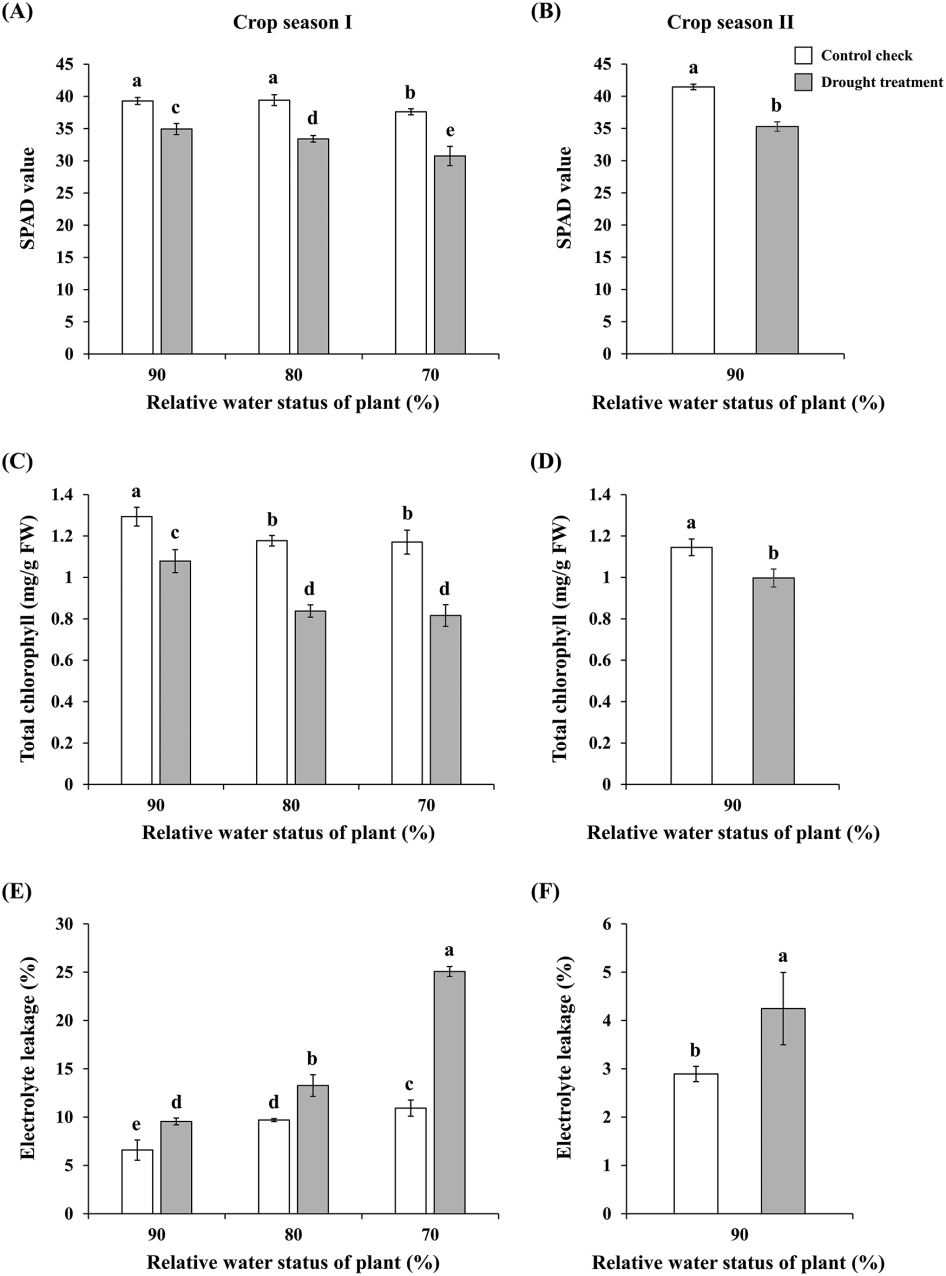
Physiological parameters of ’TN11’ under varying water status. (**a**) and (**b**) SPAD values of rice plants during the first and second cropping seasons of 2022, respectively. (**c**) and (**d**) Total chlorophyll content of rice plants during the first and second cropping seasons of 2022, respectively. (**e**) and (**f**) EL rate of rice plants during the first and second cropping seasons of 2022, respectively. The experiment was conducted independently at least three times. Error bars represent SDs. The letters indicate significant differences between water status based on the LSD post hoc test (*p* < 0.05)

Plants exhibit visible physiological changes, such as leaf yellowing and wilting, when under stress. To examine the physiological changes in rice plants under varying drought conditions, drought treatments were applied to the experimental site by withholding irrigation. The authors hypothesized that the water stressed levels of the rice plants in the experimental site would decrease to 90%, 80%, and 70% of their CP values, respectively. Furthermore, critical physiological indicators of rice under drought stress, such as SPAD value, total chlorophyll content, and EL rate, were measured to explore physiological changes caused by varying drought condition severity. The results revealed that when the water stressed levels of rice plants decreased to 90%, 80%, and 70% of CP values, their SPAD values and total chlorophyll content were substantially lower than those under CP, decreasing in proportion to increases in drought severity (Fig. 3a ~ d). By contrast, the EL rate was substantially higher than that under CP and increased as the drought severity worsened (Fig. 3e, f).

### Rice canopy temperature and thermal images under drought stress

**Fig. 4:**
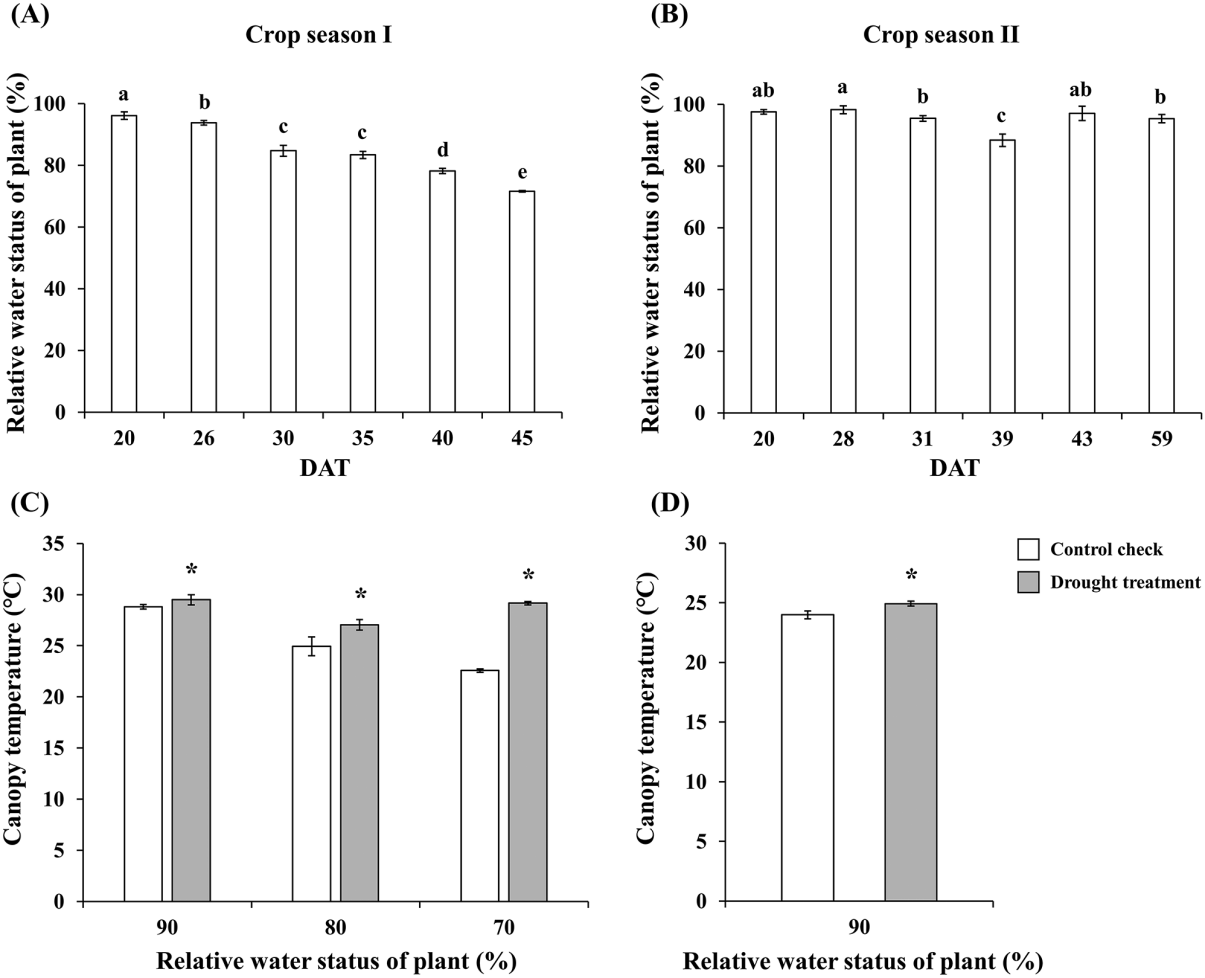
Relative water status and canopy temperatures of ’TN11’ under drought treatment in the first and second cropping seasons of 2022. (**a**) Relative water status in the first and second cropping seasons. (**b**) Canopy temperature in the first and second cropping seasons. The experiment was conducted independently at least three times. Error bars represent SDs. Asterisks (*) indicate significant differences between varying water status (Student’s t-test, *p* < 0.05). The letters indicate significant differences between samples (LSD post hoc test, *p* < 0.05). DAT: day after treatment

Outdoor field experiments are susceptible to climate fluctuations, pest infestations, and equipment malfunctions that may introduce errors or hinder data collection. In this study, during the initiation of the drought treatment period in the two cropping seasons of 2022, the relative water status of the rice plants in the first cropping season decreased to as low as 70% of CP values due to reduced rainfall during the mid-drought period (Figs. 2c and 4a). By contrast, the second cropping season experienced increased rainfall, resulting in the relative water status decreasing to only 90% of CP values (Figs. 2d and 4b).

The use of smart technologies is vital to agriculture. At least one study employed thermal imagers to detect leaf temperatures and examine their relationship with plant water status (Li et al. 2014). In the present study, when the water status of rice plants reached 90%, 80%, and 70% of CP values, thermal images were captured using the handheld thermal imager Thermo GEAR G100EX. After analyzing the thermal images based on temperature points, we observed that the canopy temperature of the rice plants was substantially higher than that of CP rice plants when water status decreased to 90% in both cropping seasons of 2022. These thermal images reveal the value of thermal imagers to detect early signs of drought stress in rice plants (Fig. 4c, d).

**Fig. 5:**
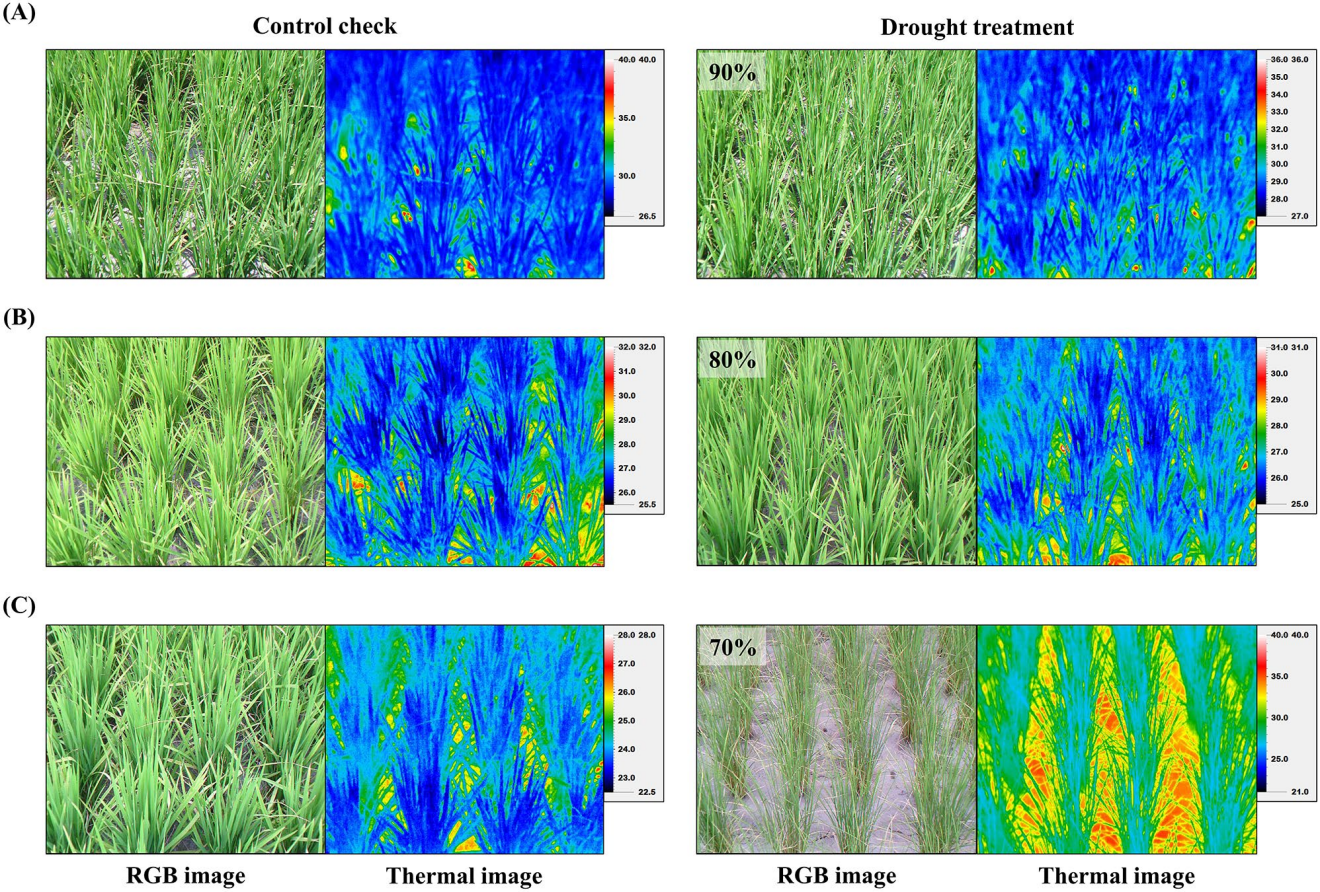
RGB and thermal images of rice variety ‘Tainan 11’ under varying water status after drought treatment. (**a**) Variations in RGB and thermal images between plants with water status of 90% of CP values and the CP control group. (**b**) Variations in RGB and thermal images between plants with a water status of 80% of CP value and the CP control group. (**c**) Variations in RGB and thermal images between plants with a water status of 70% of CP values and the CP control group

In this study, thermal imaging was employed to assess water status in rice plants in the experimental site. The imaging position was set facing the target plants at a 45-degree angle, at a distance between 1.5 and 2 m. Environmental correction was performed based on the atmospheric temperature and relative humidity. Each capture included simultaneous acquisition of RGB images and thermal images. From the imaging results, we observed that rice plants with a relative water status of 90% of CP compared with those under CP were not easily distinguishable in the RGB images. By contrast, substantial differences between the plants at these water status were evident in the thermal images (Fig. 5).

Scholars exploring the relationship between transpiration, canopy temperature, and air temperature have proposed the concept of the CWSI to integrate these factors. When soil moisture is limited, plant stomata closure reduces transpiration, leading to an increase in leaf surface temperature due to reduced evaporative cooling (Idso et al. 1981). Under drought conditions, when leaf temperature measurements are matched to air temperature measurements, the CWSI value approaches 1 as plant drought stress worsens. By contrast, the CWSI value approaches 0 as plant water status improve. To assess the feasibility of using CWSI to evaluate drought stress in rice fields, Pearson correlation coefficient analysis was conducted on CWSI, canopy temperature, water status, drought-related physiological parameters, and meteorological data for the first and second 2022 crop cycles. Regression analysis was performed using CWSI and water status data. The results revealed a positive correlation between CWSI values and water status, SPAD values, and chlorophyll content, and a negative correlation with EL rate. The correlation between water status and physiological parameters was consistent with the correlation between water status and CWSI values. Additionally, the regression model established with CWSI values and water status demonstrated satisfactory explanatory power, with a coefficient of determination (R²) of 0.8551. Therefore, CWSI can be used to effectively assess rice water status in the field and serve as a reference index for agricultural irrigation management.

## Discussion

Our study utilized handheld thermal imaging devices to capture outdoor rice fields and enable real-time assessment of rice plant water status. Additionally, due to the close associations between thermal imaging and climate conditions, a micrometeorological station was established at the site edge to monitor meteorological factors such as atmospheric temperature and relative humidity (Fig. 1b). Compared with other crops, rice typically requires more irrigation water to cultivate. Under water-deficient conditions, the appearance, chlorophyll content, EL, and water status of rice are adversely affected (Pandey and Shukla 2015; Khan et al. 2017a, 2017b). Chlorophyll is a critical factor affecting photosynthetic reactions during rice breeding, and chlorophyll levels are often used as an indicator of photosynthetic efficiency (Hu et al. 2009). Under drought conditions, rice chlorophyll is degraded and photosynthesis is inhibited, leading to a substantial decrease in chlorophyll content when measured using nondestructive chlorophyll meters such as the SPAD-502 (Swapna and Shylaraj 2017). When plants are exposed to stressors such as drought, salinity, heavy metals, or high temperatures, cell membranes are damaged, leading to ion leakage. Thus, ion leakage rates are often used as a standard to measure plant tolerance to stressors (Dasgupta et al. 2015). Studies have demonstrated that when rice is subjected to drought stress during the heading or grain filling stages, the ion leakage rate within the plant is higher than that during the tillering stage, suggesting that drought conditions severely affect the growth conditions of rice (Demidchik et al. 2014). In this study, ’TN11’ exhibited a gradual decrease in water status after irrigation cessation during the mid-tillering stage. Results revealed significant decreases in SPAD values and chlorophyll content of ‘TN11’ when the water status of the plant decreased to only 90% that were accompanied by a significant increase in ion leakage rate. Additionally, the effects of drought on physiological parameters became more apparent with increasing drought severity (Fig. 3).

**Fig. 6:**
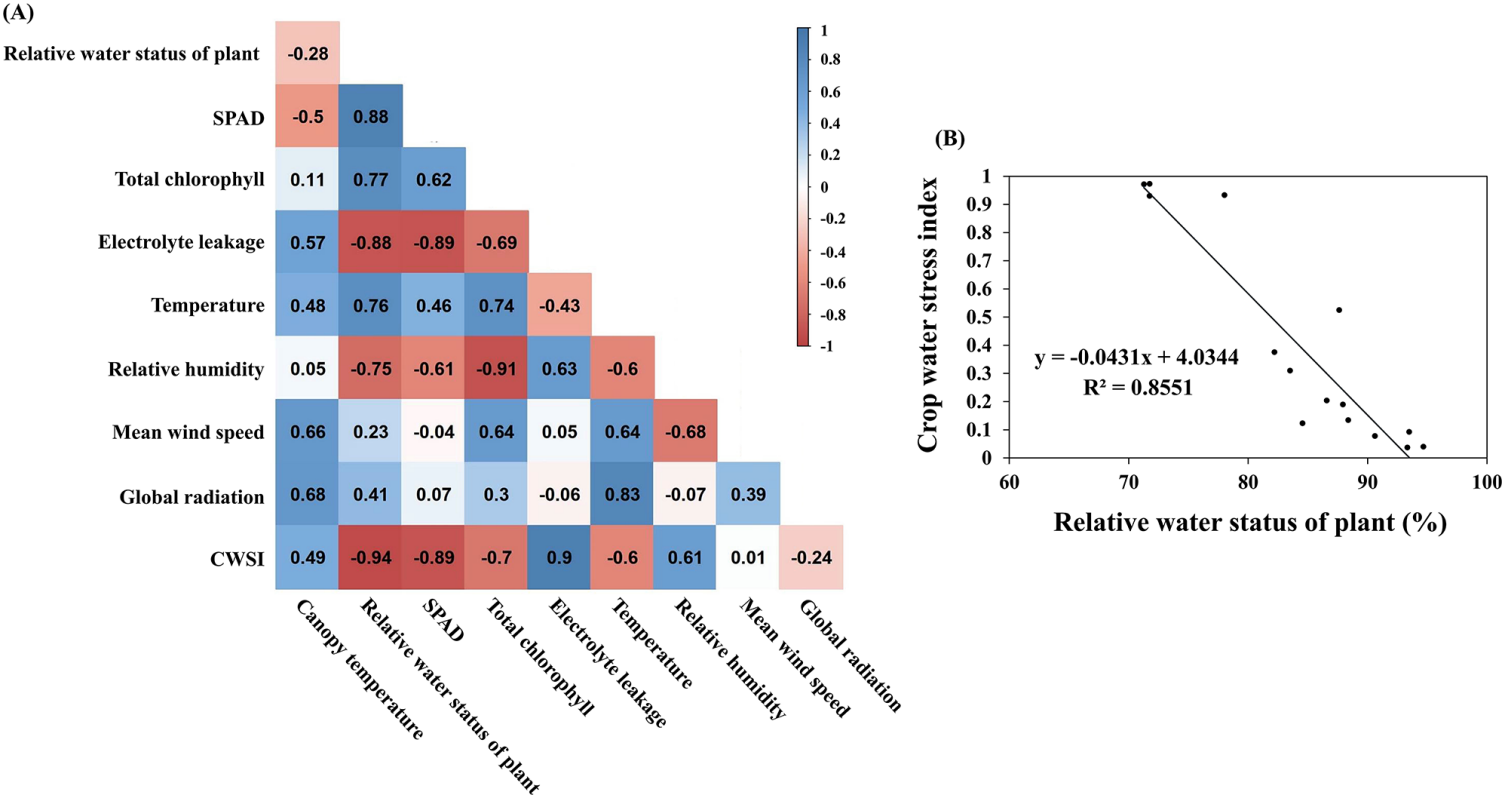
the Pearson correlation coefficient analysis and regression analysis with CWSI values and water status. (**a**), it illustrates the Pearson correlation between canopy temperature, physiological parameters, meteorological factors, and drought stress indicators for the first and second 2022 crops of ‘Tainan 11’. (**b**), it presents the regression analysis between CWSI and plant water status (RWC). The coefficient of determination (R²) is included as a measure of the strength of the correlations in the regression analysis

Leaf water content is a critical physiological parameter for assessing plant tolerance to stressors. Under normal conditions, plants facilitate water transport through the tension generated by transpiration. In response to water deficiency, plants regulate leaf stomatal closure to prevent water loss (Korres et al. 2017; Ding et al. 2018). However, the closure of stomata reduces the heat energy dissipated through water transpiration, leading to an increase in leaf temperature (Jones 2004). Studies have demonstrated a correlation between leaf temperature and the temperature difference between leaves and the atmosphere (Zhang et al. 2019). The current study’s results indicate that rice canopy temperature is significantly higher under drought stress than during CP, with substantial variations observed as early as when the plant’s water status (RWC) reaches 90% of CP values. At this stage, differences between plants maintained under CP and drought conditions are not easily distinguishable from RGB images, but can be detected from thermal images and temperature data (Figs. 4 and 5). Leaf temperature is easily influenced by shading from other plants or surrounding environmental conditions. To reveal variations in leaf temperature caused by the surrounding environment, studies have combined canopy temperature measurements from thermal imaging with environmental conditions to create stress indices as indicators of plant water status, such as the CWSI (Jones 2004; Guilioni et al. 2008). These studies reveal an inverse relationship between rice water status under drought conditions and CWSI values, with CWSI values highly correlated with stomatal conductance (Elsherbiny et al. 2021). Pearson correlation coefficient analysis in this study uncovered a high correlation between plant water status, CWSI values, and drought-related physiological parameters. Moreover, the regression analysis results for plant water status and CWSI indicated acceptable fit (Fig. 6). Our results highlight the importance of using thermal imaging and CWSI in monitoring rice drought stress and contribute to the development of smart irrigation systems that can optimize water use and enhance the sustainability of rice cultivation amidst the challenges posed by climate change and water scarcity.

## Conclusions

Rice irrigation management is critical due to the uneven rainfall distribution caused by climate change. Furthermore, farmers have traditionally relied on experience to judge rice water status or followed conventional irrigation management practices and overwatered their crops. This study employed handheld thermal imaging devices to capture RGB and thermal images of rice fields and analyzed the relationship between canopy temperature, plant water status, physiological parameters, and meteorological factors to develop a model for evaluating rice field water conditions using CWSI. The results of this study offer crucial information for field irrigation decision-making and contribute to the development of intelligent water management systems based on thermal imaging technology, enabling efficient usage and stewardship of water resources in rice cultivation.

## Electronic supplementary material

Below is the link to the electronic supplementary material.

Supplementary Material 1

## Data Availability

Data sharing not applicable to this article as no datasets were generated or analysed during the current study.
